# Evaluation of medicines dispensing pattern of private pharmacies in Rajshahi, Bangladesh

**DOI:** 10.1186/s12913-017-2072-z

**Published:** 2017-02-13

**Authors:** Shuvashis Saha, Md. Tawhid Hossain

**Affiliations:** 1 Rajshahi Medical College, University of Rajshahi, 6002 Rajshahi, Bangladesh; 2 Rajshahi Medical College Hospital, Rajshahi, Bangladesh

**Keywords:** Drug sells, Bangladesh, Pharmacy, Self medication, Drug policy

## Abstract

**Background:**

In developing country like BANGLADESH, people depend more on pharmacies due to expediency, shorter waiting time, cost reduction, availability of credit and flexible opening hours. The aim of this study was to investigate medicines dispensing patterns of the pharmacies in RAJSHAHI, BANGLADESH and to identify and analyze contribution of drugsellers and quacks in irrational drug use.

**Methods:**

This cross-sectional study was conducted during January 2016 - April, 2016 in 75 randomly selected private pharmacies including both licensed and unlicensed pharmacies of covering LAKSHMIPUR area.

**Result:**

During the whole study process, total 7944 clients visited the pharmacies under observation and 24,717 medicines were dispensed. 22.70% of all these drugs were sold without a prescription. Out of the 5610 items dispensed without prescription, 66.2% were dispensed on the request of clients themselves and 33.8% on the recommendation of a drug seller. Number of medicine in a prescription was highly variable ranging from 2 to 5 medicines per prescriptions (mean = 3.03). The average number of medicines dispensed from each of the pharmacies during the observation period was 392, varied pharmacy to pharmacy – ranging from 194 to 588. Lowest selling medicines were sedative and hypnotics and highest selling medicines were antimicrobials. The recommendation rate for antibiotics was highest for the quacks (26.48%) though the major amount of the antimicrobials (*n* = 3039, 65.83%) were dispensed on prescription. Macrolides, quinolones, metronidazoles and cephalosporins are most favourite drug of quacks, clients and pharmacists.

**Conclusion:**

Majority of medicines were dispensed irrationally without any prescription and over the counter dispensing of many low safety profile drugs was common. The results and discussion presented in this paper will be helpful to provide a baseline to redirect further studies in this area.

**Electronic supplementary material:**

The online version of this article (doi:10.1186/s12913-017-2072-z) contains supplementary material, which is available to authorized users.

## Background

Medicines are the weapons to combat disease process but may also cause serious harm when improperly used and depending on patient’s patho-physiologic factors and pharmacologic properties of the medicine this event can be as dangerous as death. Sir William Osler said “One of the first duties of the physician is to educate the masses not to take medicine” [1]. In developing countries drug monitoring system is very poor and it is very easy to buy any drug with or without prescription [2]. This inappropriate manner of medicines dispensing is one of the key element to promote irrational use of medicine [3]. Rational use of medicine is defined by the World Health Organization as ‘when patients receive medications appropriate to their clinical needs, in doses that meet their own individual requirements, for an adequate period of time, and at affordable prices’ [4]. More than 50% of all medicines prescribed, dispensed, or sold around the globe are somehow inappropriate and at the same time 50% of patients fail to take medicines appropriately [5]. Irrational use of medicines may occur in different form but self-medication and recommendation of drug by pharmacist and quacks are the most frequent forms [6]. Besides in a number of countries quality of drugs and inappropriate use of antibiotics is a growing concern [7]. From a public health viewpoint this situation needs special attention because due to these malpractices mass population become more vulnerable to excess healthcare cost, adverse drug reaction, allergic reactions, toxic poisoning, exacerbation or prolongation of critical illness, antibiotic resistance and most importantly unproductive and perilous treatment [8]. Antibiotic resistance increases cost of treatment and the poor people often have to choose between going untreated and spending huge money on drugs.

BANGLADESH is the seventh most populous country in the world and population of the country is expected to be nearly double by 2050 [9]. In BANGLADESH physicians to population ratio was 1:3600 in 2011 [10]. This critical scarcity of registered physicians creates a communication gap among physicians and patients making pharmacies a major location to seek solution to health problems. In developing countries like BANGLADESH, people depend more on pharmacies due to expediency, shorter waiting time, cost reduction, availability of credit and flexible opening hours [11, 12]. Here some drug seller also provide clinical services to patients with chronic disease even manages mild non severe trauma [13].

The aim of this study was to investigate patterns of dispensing of medicines by the pharmacies in RAJSHAHI, BANGLADESH and to identify and analyze contribution of drugsellers and quacks (persons unauthorized to practice medicine) in irrational drug use.

Though there are previous studies in BANGLADESH which demonstrated increased self medication tendency [14] and role of community pharmacists [13] but this was the first study which focused on both drug dispensing pattern of the pharmacy and tried to shed light on the contribution of pharmacies in irrational drug use.

## Methods

### Survey site

RAJSHAHI is a metropolitan city in BANGLADESH located on the northern banks of the river Padma, near the BANGLADESH-INDIA border. It is a major urban and industrial centre of North Bengal. It has an estimated population of 4,49,756. The area of the RAJSHAHI metropolitan city is 96.72 sq km [15]. The city has 4 medical colleges and more than 80 clinics and 90 diagnostic centers [16, 17]. Most of these clinics and three of the medical college hospitals are situated in LAKSHMIPUR area [16, 17]. There are more than 400 pharmacies in LAKSHMIPUR area [18]. This area was selected as major health care institutions cluster in the area and also for convenience of the study (close proximity to the study centre). The sampling frame included outlets situated in LAKSHMIPUR selling allopathic medicines and did not included hospital pharmacies and private dispensaries.

### Study design and data collection

This cross-sectional study was conducted during January 2016 - April, 2016 in 75 randomly selected private pharmacies including both licensed and unlicensed pharmacies of covering LAKSHMIPUR area from the allopathic retail pharmacy list by Ministry of Health & Family Welfare, Government of the People’s Republic of Bangladesh. The study procedure was divided into two stages. Firstly three trained data collector (medical students of Rajshahi medical college) was assigned to a randomly selected pharmacy. They interviewed the clients just after they get out from the pharmacy using a structured questionnaire (available as Additional file 1), reviewed the prescription and examined the prescription packages. The client was briefly informed by a written paper prepared by the chief investigator and written consent was taken. The process was carried out in two shifts of 3 h in the morning (9 to 12 AM) and in the afternoon business hours (4 to 7 PM) of one working day. At the end of first stage, on the same working day chief investigator interviewed the pharmacist using a structured questionnaire. The chief investigator also tried to have a short discussion with the pharmacist to understand pharmacist-client relationship. The pharmacist was also briefly informed by the chief investigator and written consent was taken. The research was carried out in 63 private pharmacies who agreed to participate (response rate =84%). Client data from other twelve pharmacies were discarded. The incomplete data provided by the clients were also discarded. In this study ‘pharmacist’ refers to the main drug seller or manager of a pharmacy.

The data collectors were given training on how to conduct the interview using the questionnaire before conduction of data collection. Actual data collection process ran from 15 January to 20 April, 2016 involving total six data collectors and the principal investigator. To ensure quality control, completed questionnaires were screened daily by the principal investigator. The client interview questionnaire included questions on prescription types, name and number of the medicines with dosage.

The pharmacist interview questionnaire included questions included questions on the pharmacist’s age, years of formal schooling with professional pharmacy qualification and work experience in a pharmacy. It also included questions on registration status of the pharmacy, history taking pattern of the pharmacist, NGO or Govt. funded training program attendance, punishment history for irrational drug selling and business hours of the pharmacy.

### Data analysis

In this paper, the term ‘hospital prescription’ is used to include medication orders issued by the physician for patients in a hospital. The term ‘private prescription’ refers to the broader category of medication orders issued by private general and specialist practitioners. Hospital or private prescriptions which were more than 30 days old were categorized as ‘old prescriptions’. It is matter of regret that currently the Directorate of Drug Administration (DDA) of Government of Bangladesh, which is the drug regulatory authority of the country, does not have any specific of OTC (Over The Counter) medicine list. For reference purpose we used OTC Medicine list by the U.S. Food and Drug Administration (USFDA). The word ‘prescription medicines’ was used to include medicines that can be sold/dispensed only with a prescription according to the USFDA.

The appropriate dosage is the dosage regimen recommended by the latest Bangladesh National Formulary (BDNF) [19]. Medicines which may have more than one indication were included in the therapeutic category of most common use. Combination medicines were analyzed by including them in different categories as appropriate. Prescriptions containing radiographic or diagnostic products were excluded from the study.

Results of the study were analyzed using Microsoft Office Excel 2007.

## Results

This study primarily enrolled 75 pharmacies but 12 pharmacists did not agreed to participate in the study (response rate =84%). During the whole study process, total 7944 clients visited the pharmacies under observation. Among this population 6313 clients bought 19,107 medicines (77.30%) with a prescription and 1631 clients purchased 5610 medicines (22.70%) without a prescription (Table 1). About 894 prescriptions were given by quacks (persons unauthorized to practice medicine). Out of the 5610 items dispensed without prescription, 3714 items (66.2%) were dispensed on the request of clients themselves and 1896 items (33.8%) on the recommendation of a pharmacist or drug seller. Additionally among 19,107 items which were dispensed with a prescription 2232 items (11.68%) were dispensed on the basis of prescription by quacks. More over 4131 (16.71%) prescription only medicines were sold without any prescription at all. Number of medicine in a prescription was highly variable ranging from 2 to 5 medicines per prescriptions (mean = 3.03). A total of 24,717 medicines were dispensed by all pharmacies during the study period. The average number of medicines dispensed from each of the pharmacies during the observation period was 392, varied pharmacy to pharmacy – ranging from 194 to 588. All therapeutic categories of drugs were sold by all four category of request. Sales of medicine according to therapeutic category reveal that lowest selling medicines were sedative and hypnotics and highest selling medicines were antimicrobials (Table 2). The recommendation rate for antibiotics was highest for the quacks (26.48%) (Table 2) though the major amount of the antimicrobials (*n* = 3039, 65.83%) were dispensed on prescription (Table 3). Anti-infectives were found to be the most recommended medicines in all groups. Table 4 shows antibiotic agents dispensing according to generic group. Macrolides, quinolones, metronidazoles and cephalosporins are most favourite drug of quacks, clients and pharmacists. Interestingly pharmacists and clients did not ask for any carbepenems at all but quacks and doctors did. Table 5 shows dispensing pattern of antimicrobials. It shows that major portion of antimicrobials sales were inappropriate (client group, 95.06%; pharmacist group, 89.57%; quack group, 84.26%). Lots of client took one or two tablets/capsules as one or two day’s supply.

**Table 1:** Medicine dispensed at pharmacies

Type of dispensing		Number	Subtotal
With prescription	Private prescription	5811 (30.42%)	19,107 (77.30%) Prescription medicine-(13,311) OTC medicine-(5796)
	Hospital prescription	8979 (46.99%)	
	Old prescription	2085 (10.91%)	
	Prescription from quacks (without valid license to practice)	2232 (11.68%)	
Without prescription	Request by client (self medication)	3714 (66.2%)	5610 (22.70%) Prescription medicine-(4131) OTC medicine-(1479)
	Recommended by pharmacist	1896 (33.8%)	
Total			24,717 (100%)

**Table 2:** Medicine dispensed by therapeutic category

Category	Prescription		Requested by client		Recommended by pharmacist		Prescription from quacks		Total	
	*n*	%	*n*	%	*n*	%	*n*	%	*n*	%
Anti infectives	3039	18.01%	963	25.93%	489	25.79%	591	26.48%	5082	20.56%
Medicines for GIT	1741	10.32%	444	11.95%	201	10.60%	183	8.20%	2569	10.39%
Medicines for CVS	1531	9.07%	378	10.18%	144	7.59%	189	8.47%	2242	9.07%
Medicines for CNS	2056	12.18%	132	3.55%	84	4.43%	123	5.51%	2395	9.69%
Medicines for respiratory system	1846	10.94%	477	12.84%	249	13.13%	336	15.05%	2908	11.77%
Vitamins and nutritional suppliments	1159	6.87%	279	7.51%	162	8.54%	219	9.81%	1819	7.36%
Analgesics and antipyretics	1408	8.34%	468	12.60%	288	15.18%	201	9.01%	2365	9.57%
Anti-diabetics	1907	11.30%	246	6.62%	99	5.22%	96	4.30%	2348	9.50%
Sedative and hypnotics	1513	8.97%	159	4.28%	114	6.01%	168	7.53%	1954	7.91%
Miscellaneous	675	4.00%	168	4.52%	66	3.48%	126	5.65%	1035	4.19%
Total	16,875		3714		1896		2232		24,717	
Prescription medicine: 17,442						OTC medicine: 7275

**Table 3:** Anti infective agents dispensed

Class	Prescription	Requested by client	Recommended by pharmacist	Prescription from quacks	Total (%)
Antibiotics	1554	636	201	309	2700 (53.13%)
Antifungals	369	39	36	93	537 (10.56%)
Antiamoebics	231	51	57	54	393 (7.73%)
Antihelminthics	198	108	69	36	411 (8.09%)
Topical antimicrobials	687	129	126	99	1041 (20.49%)
Total (%)	3039 (65.83%)	963 (16.11%)	489 (8.18%)	591 (9.88%)	5082 (100%)

**Table 4:** Antibiotic agents dispensing according to generic group

Class	Prescription	Requested by client	Recommended by pharmacist	Prescription from quacks	Total
Penicillin	60	9	6	6	81
Cephalosporins	312	143	54	48	557
Carbapenems	31	0	0	9	40
Tetracyclin	147	9	9	24	189
Aminoglycosides	93	15	3	24	135
Quinolones	369	132	30	61	592
Metronidazole	129	76	24	21	250
Sulfonamide	30	39	3	15	87
Macrolides	390	193	69	93	745
Others	24	0	0	0	24
				Total	2700

**Table 5:** Dispensing pattern of antimicrobials

Type of dispensing	Dosage	Number	Percentage
Prescription	Prescribed dose	1877	61.77%
	Inadequate dosage	1162	38.23%
	Total	3039	
Requested by client	Full dosage	39	4.04%
	Inadequate dosage	924	95.06%
	Total	963	
Recommended by pharmacist	Full dosage	51	10.43%
	Inadequate dosage	438	89.57%
	Total	489	
Prescription from quacks	Full dosage	93	15.74%
	Inadequate dosage	498	84.26%
	Total	591	
All four types	Full dosage	2060	40.53%
	Inadequate dosage	3022	59.46%
	Total	5082	

Table 6 shows other service provided by pharmacists. Most of the pharmacists inject IV or IM drug to the patients, measure blood pressure and measure blood glucose by portable machine. Maximum findings presented in Table 7 are alarming. 58.74% pharmacy did not have valid and updated registration while 96.83% percent of the pharmacist recommended medicine taking inadequate history. 53.96% of drug seller did not have any professional qualification in pharmacy and they mainly relied on combined knowledge pool of work experience, medical representatives, distributors and doctors. Despite of these reckless practices none of them ever received any punishment for irrational drug selling.

**Table 6:** Other service provided by pharmacists

Screenings	Responses	
	Yes	No
Blood pressure measurement	37	26
Blood sugar measurement	34	29
Dressing	21	42
Nebulization	32	31
stitches with suture materials	39	24
Inject IV or IM injections to patients	56	7

**Table 7:** Basic characteristic distribution of the pharmacists

Variable	Frequency (%)
Mean Age in years (SD)	34 ± 7.5
Does this pharmacy valid registration	Yes (41.26%)
	No (58.74%)
Do you take history systematically before recommending medicine?	Yes (3.17%)
	No (96.83%)
Do you provide the patients necessary information regarding possible side effect of the drugs every time?	Yes (4.76%)
	No (95.24%)
Did you ever had any training on pharmacy maintenance from any govt. or NGO based organization?	Yes (38.09%)
	No (61.90%)
Did you ever participated in any health education program organized by any govt. or NGO based organization?	Yes (7.93%)
	No (92.06%)
Did you ever receive any punishment for irrational drug selling?	Yes (0%)
	No (100%)
Professional qualification	
Basic training in pharmacy	23 Responses (36.50%)
Diploma in pharmacy	5 Responses (9.93%)
Bachelor of pharmacy	1 Response (1.58%)
No education on pharmacy	34 Responses (53.96%)
Mean year of schooling (SD)	10.9 ± 1.85
Mean work experience (SD)	10.53 ± 4.97
Source of current drug knowledge	
On the job experience	8 Responses (12.70%)
Medical representatives	6 Responses (9.52%)
Doctor	1 Response (1.58%)
Distributors	4 Responses (6.34%)
Combined	44 Responses (69.84%)
Mean opening hours of pharmacy	13.58 ± 4.72

The mean age of drug sellers was 34 years (SD 7.5) Mean years of formal schooling was 10.91 years (SD 1.85). The mean work experience of drug sellers was 8.53 years (SD 4.97). The mean work hour of the pharmacies were 13.58 h (SD 4.72).

## Discussion

Modern Bangladesh emerged as an independent nation in 1971 when average life expectancy at birth was 47.05 years and now it is 71.6 according to World Bank data published in 2014 [20]. Continuous development in healthcare facilities improved the condition. Still healthcare sector in BANGLADESH has lots of things to develop. The current study assessed the scope for improvement in rational drug distribution policy. To our knowledge, this was the first formal study to address drug dispensing pattern of private pharmacies in BANGLADESH.

In BANGLADESH self medication is a rising problem. Self-medication is becoming prominent as it saves time and money. Poor people may self-medicate to save the costs for physician’s consultancy and also save money by not taking a full course or by using less priced below-standard products. Quacks of BANGLADESH generally do not charge consultation fees; Drug selling profit is their main source of income [21]. So recommendation of drugs can be frequently driven by financial concerns and not by rational therapeutic choice. The rapid access to mobile phones and the internet is contributing to promotion of self medication as drug information is now widely available though its authenticity and appropriateness is not verified. Previous studies showed that people are engaged in self medication though the rate is variable among different education group [14, 22]. Current study revealed that 22.7% of all medicines were sold without any prescription. The percentage is quite consistent with the data of a former study done in the same area on self medication of antibiotics [14]. There are lots of factors behind this high rate of self medication. In this study we found that antimicrobials are highest selling drug contributing 20.56% of all drugs. Though anti-infective recommendation rate is lowest in valid prescription group (18.01%) but almost every 48 valid prescriptions out of 100 had at least one antibiotic, which is too high. On the other hand every 62 prescription from quacks out of 100 had at least one antibiotic. Out of every 100 client 86 client bought at least one antibiotic either due to pharmacist’s recommendation or as self medication.

Health workers often do not follow good practice guidelines. Knowledge is not the only key element here. Several factors influence this deviation from best practice. In some areas proper diagnostics facilities like blood culture are unavailable, doctors use antibiotics as a frontline treatment. Though marking such practices as irrational is questionable especially when the evidence regarding risks of overuse and the effectiveness of interventions are unknown. Considering poor economic conditions of the patients they often avoid culture and sensitivity test rather they prescribe an empirical antibiotic therapy. Doctors and patients adjust what can be afforded [23]. Qualified physicians often blame patient demand for irrational prescription. There is a reputational risk as in communities people judge the quality of the physicians by their willingness to recommend diagnostic test and ‘powerful’ medicines on demand [24, 25]. In BANGLADESH employees of drug-distribution or production companies, who are known as medical representatives act as a source of information for both registered and unregistered physician, offer financial inducements and many other ‘gifts’ during their visit to the physicians. These information cannot be regarded as impartial [26] and there is lack of clarity regarding what kind of financial deals are made during these visits.

Common people believe antibiotics can treat and eradicate any infections irrespective of their origin which was found in previous studies [14, 22]. Antibiotics are actually a victim of their own advantages; they are low cost and easy to consume. Common people regard them as ‘powerful’ medicines which can treat lots of ailments. 59.46% of all antimicrobials were bought in inadequate dosage. This irrational use of antimicrobials may lead to creation of super infection or mask the symptoms of infection making the actual diagnosis process complex. Inadequate antimicrobial treatment is an important factor in the emergence of infections due to antibiotic-resistant bacteria [27] and rise of antimicrobial resistance in a community increasing total healthcare expenditure [28]. Past experience, familiarity and better efficacy of a drug is one of many reasons for selecting a particular antibiotic for self medication. Leftover antibiotic at home either as a result of over-prescription or patient’s non-compliance with a course of treatment is also another important factor [29].

In every country the pharmaceuticals industry is controlled by a regulatory authority. The Directorate of Drug Administration (DDA) under the Ministry of Health & Family Welfare, Government of the People’s Republic of Bangladesh, is the drug regulatory authority of the country. Drug regulation in BANGLADESH is patchy. Currently there are no over the counter (OTC) drug list approved by the authority. That means the pharmacies have legal right to sell registered drugs to anyone without any prescription even drugs for cardiovascular diseases and diabetes. In BANGLADESH, quality control systems of drugs are weak and a great fraction of drugs are counterfeit or sub-standard [30].

In recent years increased pharmaceuticalization and commodification of health has taken place. As a result people are much more motivated to ‘reach for the pill’ at first sign of illness [31]. Now-a-days common people have decreased tolerance level for symptoms and at a same time greater familiarity with drugs and pharmacists. The pharmacists also have important role in fostering self medication. This study reveals that among 5610 medicines, which were sold without prescription, 33.8% were sold on recommendation by the pharmacist and 66.2% on the basis of client request. It appears that client request is the major contributor but pharmacists are also responsible. It appears that fairly a lot of people ask for advice of medicine seller on common ailments or just directly ask for previously known medicine. Such clients often have unrealistic expectations including immediate resolution of symptoms. Therefore, pharmacy personnel also tend to recommend medicines with dramatic action and lucrative profit irrespective of medicine’s safety profile. This study revealed that 96.83% of medicine sellers recommend medicine without asking for complete history even drug allergy history. This desperate tendency of drug selling can be explained as ill-trained medicine vendors are always too eager to recommend medicines [32]. Moreover 58.74% of pharmacies are operating without valid and up-to-date license, 53.96% medicine sellers don’t have any formal education in pharmaceutical science, 61.90% never had any training on pharmacy maintenance from any govt. or NGO, 92.06% of them never participated in any health education program organized by any govt. or NGO and In spite of this critical condition none of the sellers ever received any punishment for irrational drug selling. To interpret the findings properly consideration of socioeconomic contexts and perceptions of the pharmacists are important which we learned during our face to face informal interview with the pharmacists or main drug sellers. In BANGLADESH drug market is expending and highly competitive. The growth in number of pharmacies in urban BANGLADESH can be attributed to the promotional activities of pharmaceutical companies and lucrative profit offered by them. From a strict business viewpoint pharmacy is a profitable venture to invest. Such investors often care for profit but not for the pharmacies’ public health impact. They are also not interested to abide by the law of land as punishment is rare. Sales competition among pharmacies is another notable factor in areas where pharmacies clustered together, especially beside large hospital and clinics. In these places competition between pharmacies is sometimes so intense that they hire ‘agents’ to persuade patients to buy medicines from a particular pharmacy by offering cash discount. These agents receive their commission for having a successful hunt. This business venture viewpoint also explains why drug vendors are not interested in formal education or training in pharmacy. Nowadays every medicine comes in prepackaged bottles and strips with label containing company name, brand name and ingredients. Pharmacists just have to take the medicines out of shelves and give it to the client. They think experience is more important than formal education in pharmacy business. Average work experience of the pharmacists we interviewed was 10.53 (SD 4.97). Though we only interviewed main drug seller of a particular pharmacy, drug seller’s assistants may have variable work experience. 95.24% of the druggists don’t aware the patients regarding possible side effect of the drugs. During our study period in an average each pharmacy took approximately less than 4 min to serve a client. Time is very short to have detailed interaction with the client.

Interestingly in spite of their lack of knowledge they know about common indication of a common drug from their experience but don’t know about its contraindications and side effect. For example they know streptomycin is for infection, losartan potassium is for blood pressure but they don’t know streptomycin can cause ototoxicity. This can also be explained by their motive to recommend medicine to increase profit knowing side effects do not add anything to the business. Even during face to face interview many pharmacists spontaneously expressed that they think it is not their duty to provide information on how medicines should be taken, possible drug reactions and when they are contraindicated. They think prescribing doctor should do that. The study also found that majority of the drug sellers depend on drug company materials for gathering knowledge. These materials often emphasise on all possible uses of the product avoiding cost-effectiveness and potential therapeutic risks [33].

The study revealed that pharmacists are offering various clinical services like blood pressure measurement, capillary blood sugar measurement and even providing stitches with suture materials as an extra source of money. This finding is consistent with previous report [34] but the efficiency of drug vendors to perform these clinical works and associated potential risks still need to be investigated.

This study reflect the problems in drug regulation with the irrational drug dispensing by the drug sellers with their lack of knowledge and training but it had some limitations. Some questions were very sensitivity to answer so we took **a**sking indirect questions approach and tried normalisation of the sensitive issue before asking. Even after that we can’t be sure that all answers given by drug sellers reflect actual situations. We also couldn’t verify the presence of counterfeit or fake drugs in stores using questionnaire or interview method due to this problem, though we intended to do that. We had the sample size of only 63 pharmacies and we selected them in an area where we have better logistic supply. Such convenience sampling of the area may have compromised our study with a selection bias. To minimize the bias we selected the pharmacies as random as possible. The study was based on a small urban area. We believe that there can be an urban–rural variation in medicine dispensing pattern of pharmacies. So multi centered or national level study is necessary to draw final conclusions.

## Conclusion

The study documents that majority of medicines were dispensed irrationally without any prescription and over the counter dispensing of many low safety profile drugs was common. Government need to take educational and regulatory interventions to improve knowledge and professional behavior of pharmacists, pharmacy assistants and drug sellers. Health education is also essential for common people to prevent self-medication. The policy maker especially Drug Administration of Bangladesh needs to build pertinent regulatory policies and ensure their implementation. Bangladesh Medical & Dental Council (BM&DC) also needs to take action against quacks to prevent maltreatment of patients. The results and discussion presented in this paper will be helpful to provide a baseline to redirect further studies in this area.

## Additional file

Additional file 1: Questionnaire for clients and Questionnaire for drug sellers. (DOCX 17 kb)

## Abbreviations

BDNF: Bangladesh National Formulary
BM&DC: Bangladesh Medical & Dental Council
DDA: Directorate of Drug Administration
NGO: Non Government Organization
OTC: Over The Counter
SD: Standard Deviation
USFDA: U.S. Food and Drug Administration
