# Supplementary material for: Evaluation of medicines dispensing pattern of private pharmacies in Rajshahi, Bangladesh
Source: BMC Health Serv Res. 2017 Feb 13;17:136. doi: 10.1186/s12913-017-2072-z (PMC5307842; doi:10.1186/s12913-017-2072-z)
Supplement: Additional file 1: — Questionnaire for clients and Questionnaire for drug sellers. (DOCX 17 kb) [file 12913_2017_2072_MOESM1_ESM.docx]

**Questionnaire for clients:**

(Please tick(√) on your answer in the box)

1. By whom the medicine was prescribed:

| Private prescription |  |
| --- | --- |
| Hospital prescription |  |
| Old prescription |  |
| Prescription from quacks (without valid license to practice) |  |
| Request by client (self medication) |  |
| Recommended by pharmacist |  |

2. Therapeutic category of Medicines: (Put numbers in the box)

| Anti infectives |  |
| --- | --- |
| Medicines for GIT |  |
| Medicines for CVS |  |
| Medicines for CNS |  |
| Medicines for respiratory system |  |
| Vitamins and nutritional supplements |  |
| Analgesics and antipyretics |  |
| Anti-diabetics |  |
| Sedative and hypnotics |  |
| Miscellaneous |  |
| Total |  |

3. Type of Anti-microbial agents (if any) (Multiple selections possible):

| Antibiotics |  |
| --- | --- |
| Antifungals |  |
| Antiamoebics |  |
| Antihelminthics |  |
| Topical antimicrobials |  |

4. Mark the antibiotic agents according to generic group (if any) (Multiple selections possible):

| Penicillin |  |
| --- | --- |
| Cephalosporins |  |
| Carbapenems |  |
| Tetracyclin |  |
| Aminoglycosides |  |
| Quinolones |  |
| Metronidazole |  |
| Sulfonamide |  |
| Macrolides |  |
| Others |  |

5. Amount of antimicrobial prescribed doses: Write Y for yes, N for No

| Full dosage |  |
| --- | --- |
| Inadequate dosage |  |

**Questionnaire for drug sellers:**

1. Other service provided by your Pharmacy:

|  | Please tick(√) on your answer | |
| --- | --- | --- |
|  | Yes | No |
| Blood pressure measurement |  |  |
| Blood sugar measurement |  |  |
| Dressing |  |  |
| Nebulization |  |  |
| stitches with suture materials |  |  |
| Inject IV or IM injections to patients |  |  |

7. Basic characteristic distribution of the pharmacists:

| Questions | Answer |
| --- | --- |
| 1. Age in years | Please write above |
| Write Y for yes, N for No for following questions |  |
| 1. Does this pharmacy has valid registration |  |
| 1. Do you take history systematically before recommending medicine? |  |
| 1. Do you provide the patients necessary information regarding possible side effect of the drugs every time? |  |
| 1. Did you ever had any training on pharmacy maintenance from any govt. or NGO based organization? |  |
| 1. Did you ever participated in any health education program organized by any govt. or NGO based organization? |  |
| 1. Did you ever receive any punishment for irrational drug selling? |  |
| 1. Professional Qualification | Please tick(√) below on your qualification |
| - Basic Training in pharmacy |  |
| - Diploma in pharmacy |  |
| - Bachelor of Pharmacy |  |
| - No education on pharmacy |  |
| 1. Years of schooling | Please write here |
| 1. Work experience | Please write here |
|  |  |
| 1. Source of current drug knowledge | Please tick(√) on your answer |
| - On the job experience |  |
| - Medical representatives |  |
| - Doctor |  |
| - Distributors |  |
| - Combined |  |
| 1. Mean opening hours of pharmacy | Please write here |
